# Development and Validation of Equations for Predicting the Metabolizable Energy Value of Double-Low Rapeseed Cake for Growing Pigs

**DOI:** 10.3390/ani11041168

**Published:** 2021-04-19

**Authors:** Lu Wang, Qile Hu, Peili Li, Changhua Lai, Defa Li, Jianjun Zang, Shouqing Ni

**Affiliations:** 1 State Key Laboratory of Animal Nutrition, College of Animal Science and Technology, China Agricultural University, Beijing 100193, China; wanglucau@163.com (L.W.); huqile44@163.com (Q.H.); peilil@126.com (P.L.); laichanghua999@163.com (C.L.); defali@cau.edu.cn (D.L.); 2 Shandong Provincial Key Laboratory of Water Pollution Control and Resource Reuse, School of Environmental Science and Engineering, Shandong University, Qingdao 266237, China

**Keywords:** double-low rapeseed cake, metabolizable energy, prediction equation, caloric efficiency, growing pig

## Abstract

**Simple Summary:**

Double-low rapeseed cake could be a cost-effective feed resource for swine, providing amino acids and energy. Accurate evaluation of the energy values of different double-low rapeseed cakes is of great significance to economically and effectively formulate diets when double-low rapeseed cakes are used as an alternative ingredient in diets fed to pigs. Therefore, two experiments were conducted to develop and validate an equation to predict the metabolizable energy of double-low rapeseed cake for growing pigs based on chemical compositions. Results indicated that the best-fit prediction equation for metabolizable energy (MJ/kg) was 9.33 − 0.09 × neutral detergent fiber −0.25 × crude fiber + 0.59 × gross energy (R^2^ = 0.93). Increasing levels of double-low rapeseed cake linearly reduced apparent total tract digestibility of nutrients, but did not affect growing performance and caloric efficiency of metabolizable energy of growing pigs fed diets balanced for standardized ileal digestibility Lys/metabolizable energy ratio. The best-fit prediction equation in this experiment can be used to accurately estimate the metabolizable energy value of double-low rapeseed cake fed to growing pigs under practical conditions.

**Abstract:**

The study was conducted to develop and validate an equation to predict the metabolizable energy (ME) of double-low rapeseed cakes (DLRSC) for growing pigs based on their chemical compositions. In Experiment 1, 66 growing pigs (initial body weight 36.6 ± 4.1 kg) were allotted randomly to a completely randomized design with 11 diets. The diets included a corn–soybean meal basal diet and 10 test diets containing 19.22% DLRSC supplemented at the expense of corn, soybean meal, and lysine. Neutral detergent fiber (NDF), crude fiber (CF), and gross energy (GE) were the best predictors to determine ME. The best-fit prediction equation of ME (MJ/kg) was ME = 9.33 − 0.09 × NDF − 0.25 × CF + 0.59 × GE (R^2^ = 0.93). In Experiment 2, a total of 144 growing pigs (initial body weight 29.7 ± 2.7 kg), with six pigs per pen and six pens per treatment, were assigned randomly to four treatments in a completely randomized block design for a 28-day feeding trial. A corn–soybean meal basal diet was prepared, and three additional diets were formulated by adding 7%, 14%, and 21% DLRSC to the basal diet at the expense of soybean meal. All diets were formulated to provide equal standardized ileal digestibility (SID) Lys/ME ratio and SID essential amino acids/SID Lys ratio. Increasing dietary levels of DLRSC had no effect on average daily feed intake, average daily gain, and feed-to-gain ratio. The caloric efficiency of ME (31.83, 32.44, 31.95, and 32.69 MJ/kg, respectively) was not changed by increasing the dietary concentration of DLRSC. Increasing dietary levels of DLRSC linearly reduced (*p* < 0.05) the concentrations of triiodothyronine and tetraiodothyronine in serum, as well as apparent total tract digestibility of DM, GE, crude protein, acid detergent fiber, and organic matter of the diet. In conclusion, the ME prediction equation obtained in Experiment 1 accurately estimates the ME value of DLRSC fed to growing pigs.

## 1. Introduction

Rapeseed is the second most abundant oilseed produced after soybean in the world, with production of more than 68 million tons in 2019 [1]. Double-low rapeseed cake (DLRSC), a co-product of expeller-press extruded double-low rapeseed to produce oil, is an attractive, cost-effective feed resource for animals [2]. Double-low rapeseed cake is produced without solvent extraction, resulting in high residual oil content (10–15%) in DLRSC [3]. Hence, DLRSC has a high energy value and may be a valuable source of amino acids (AA) in swine diets. Double-low rapeseed cake has been widely used in swine feed formulation to reduce diet cost and has no negative effects on the growth performance of pigs [4,5].

Energy value for DLRSC varies greatly due to its diverse chemical composition [6]. In vivo measurement of energy value is not only time consuming and expensive, but the measured values are only applicable to the specific samples evaluated in the experiment. Prediction equations that estimate the energy value based on chemical composition can be used to rapidly and accurately estimate energy value of feed ingredients [7,8]. However, to our knowledge, there is no available information on the prediction of energy content in DLRSC for growing pigs. Furthermore, for these equations to be effective, validation studies using animal tests are needed [8,9]. There is, however, no clear agreement on which method to use to verify the accuracy of a prediction equation. Commonly used methods include caloric efficiency [10], cross-validation [11], and repeated experimentation [12,13] methods. In this experiment, the caloric efficiency method was used to verify the accuracy of the ME prediction equation.

We hypothesized that the energy prediction equations can be established and used to accurately predict the metabolizable energy (ME) of DLRSC. Therefore, the objective of this study was to: (1) develop prediction equations for ME based on the chemical compositions and energy values of 10 different DLRSC samples previously analyzed in our laboratory; and (2) verify the accuracy of the best-fit prediction equation for ME using the caloric efficiency approach.

## 2. Materials and Methods

All protocols used in our experiments were reviewed and approved by the Institutional Animal Care and Use Committee of China Agricultural University (Beijing, China; No. AW52101202-2-2). Two experiments were conducted at the FengNing Swine Research Unit of China Agricultural University (Academician Workstation in Chengdejiuyun Agricultural and Livestock Co., Ltd., Hebei, China).

### 2.1. Experiment 1

#### 2.1.1. Experimental Design, Dietary Treatments, Sample Collection, Chemical Analysis, and Calculation

Experiment 1 was conducted to determine the ME prediction equations. Details about the experimental design, dietary treatments (Table 1), sample collection, chemical analysis, and calculations were the same as those described in Experiment 1 in our previous paper reporting the energy content of DLRSC fed to growing pigs [6]. Therefore, readers are advised to refer to that paper for more information. Chemical compositions and ME values of DLRSC from a published study (Supplemental Table S1) [6] were used to develop the ME prediction equations.

#### 2.1.2. Statistical Analyses 

Data were analyzed statistically using SAS 9.4 (SAS Inst. Inc., Cary, NC, USA). Normal distribution and homogeneous variance of data were verified using the UNIVARIATE procedure of SAS, and no outliers were identified. In Experiment 1, prediction equations for ME in the DLRSC samples were determined using PROC REG of SAS. Stepwise regression analyses were performed using data obtained from the Supplementary Table S1, with ME values as a dependent variable and chemical compositions of DLRSC as independent variables. The stepwise selection procedure begins by first including the regressor variable with the highest simple correlation to the dependent variable. As each regressor is entered into the model, the partial correlation coefficients of the remaining candidate regressors are calculated to adjust for the effect of each selected variable on the dependent variable. The candidate regressor with the largest partial correlation coefficient then enters the model. At each step, the regressors in the model are reevaluated for significance and may be removed if they exceed the criteria for entry. The process is repeated until no further candidate regressors meet the criteria for entry or elimination [14]. In the current study, entry and elimination criteria were set at *p* ≤ 0.15. The best regression models were determined using multiple criteria analyses in which the R^2^, Akaike information criterion (AIC), and root mean square error (RMSE) of the model were considered. The equation with the greatest R^2^ and the lowest RMSE and AIC were chosen as the best-fit model. 

### 2.2. Experiment 2

#### 2.2.1. Experimental Design and Dietary Treatments

Experiment 2 was conducted to determine the accuracy of a prediction equation for ME content generated from the chemical composition of DLRSC in Experiment 1. A total of 144 growing pigs (Duroc × Landrace × Large White; initial body weight (BW) 29.7 ± 2.7 kg) were assigned randomly to 4 treatments in a completely randomized block design for a 28-d feeding trial. Each treatment diet was fed to 6 replicate pens with 6 pigs (3 barrows and 3 gilts) per pen. The corn, soybean meal, and DLRSC used in this experiment were the same as those reported previously [6]. The chemical composition of DLRSC was analyzed, and values used in the best-fit prediction equation obtained in Experiment 1 were used to calculate the ME of DLRSC (Table 2). The ME of other ingredients were those published by the NRC [15]. The basal diet was a corn and soybean meal diet (Table 3). Three additional diets were formulated by adding 3 levels of DLRSC (7%, 14%, and 21%) to the basal diet to mainly replace soybean meal. The ratio between standardized ileal digestibility (SID) Lys and ME values was the same among the four diets. The ratio between SID essential amino acids and SID Lys was kept constant and met the ideal protein profile. All diets were formulated to meet or exceed the estimated nutrient requirements for growing pigs (with BW of 25–50 kg) as recommended by the NRC [15], and the analyzed composition of the experimental diets in Experiment 2 is summarized in Table 3. The pigs were housed in pens with half woven mesh and half cement floor. Pigs had free access to feed and water throughout the 28-day experimental period. Room temperature was maintained between 22 and 24 °C.

#### 2.2.2. Sample Collection 

Samples of the diets and DLRSC were collected and stored at 4 °C until analyzed. All pigs were individually weighed using an electronic weighbridge with fence (special for grower-finisher pigs; Shirun Industrial Co., Ltd., Shanghai, China) at days 0 and 28. Feed added was weighed and recorded per pen throughout the whole experimental period. Leftover feed was weighed and recorded per pen at the end of the experiment. Feed spillage was collected before feeding to be dried, weighed, and recorded. All collected data were used to calculate pen average daily feed intake (ADFI), average daily gain (ADG), and feed-to-gain ratio (F/G). During the last week of the experiment, chromium oxide (Cr_2_O_3_; 0.30%) was added to all diets as an inert indicator to calculate apparent total tract digestibility (ATTD) of nutrients. On days 26 and 27 of the experiment, approximately 150 g of representative feces was collected from each pen, and the fecal samples were stored at −20 °C. The 2-day fecal samples from each pen were pooled and then oven-dried at 65 °C for 72 h. All samples were ground to pass through a 1-mm screen before further chemical analysis. On the morning of day 28, one pig from each pen was selected randomly to provide a blood sample (8 mL) from the jugular vein after overnight fasting. Blood was collected into anticoagulants-free Vacutainer tubes (Becton Dickinson Vacutainer Systems, Franklin Lakes, NJ, USA). Serum was obtained after centrifugation (Biofuge22R; Heraeus, Hanau, Germany) at 3000× *g* for 15 min and stored at −20 °C for further analyses. 

#### 2.2.3. Chemical Analysis and Calculation

All DLRSC samples, experimental diets, and fecal samples were analyzed for dry matter (DM; method 930.15) [16], crude protein (CP; method 990.03) [16], ether extract (EE) [17], crude ash (method 942.05) [16], crude fiber (CF; method 978.10) [16], acid detergent fiber (ADF), neutral detergent fiber (NDF), and gross energy (GE). The ADF and NDF were determined using F57 filter bags and fiber analyzer equipment (Fiber Analyzer; Ankom Technology, Macedon, NY, USA) according to the procedure of Van Soest et al. [18] with a slight modification. The NDF was analyzed using heat-stable α-amylase and sodium sulfite without correction for insoluble ash. The GE were analyzed using an Automatic Isoperibol Oxygen Bomb Calorimeter (Parr 6300 Calorimeter; Parr Instrument Company, Moline, IL, USA). All DLRSC samples were analyzed for calcium (method 968.08) [16], total phosphorus (method 946.06) [16], and total glucosinolates [19]. Experimental diets and fecal samples were analyzed to determine concentrations of chromium using a polarized Zeeman Atomic Absorption Spectrometer (Hitachi Z2000, Tokyo, Japan) after nitric acid-perchloric acid wet ash sample preparation. The concentrations of triiodothyronine (T3) and tetraiodothyronine (T4) in serum were determined using enzyme-linked immunosorbent assay kits (Beijing Huaying Bioengineering Institute, Beijing, China) and an automatic biochemical analyzer (Hitachi 7160, Hitachi High-Technologies Corporation, Japan).

The organic matter (OM; %) was calculated as DM-ash. The caloric efficiency of ME (MJ/kg) was calculated as ADFI (kg/d) × diet ME content (MJ/kg)/ADG (kg/d). The ATTD (%) of nutrients were calculated as (1 − 1 × (concentration of Cr_2_O_3_ in diet × concentration of target nutrient in feces)/(concentration of Cr_2_O_3_ in feces × concentration of target nutrient in diet)) × 100. 

#### 2.2.4. Statistical Analyses

Data were analyzed statistically using SAS 9.4 (SAS Inst. Inc., Cary, NC, USA). Normal distribution and homogeneous variance of data were verified according to the method of Experiment 1, and no outliers were identified. In Experiment 2, data were analyzed statistically using the MIXED procedure of SAS with each pen as the experimental unit. The statistical model had dietary treatment as a fixed effect and block as a random effect. Orthogonal polynomial contrasts were used to determine effects of increasing amount of DLRSC in the diet on growth performance, ME caloric efficiency, serum parameters, and nutrient digestibility. Treatment means were calculated using the LSMEANS statement and differences were considered significant if *p* < 0.05. 

## 3. Results

### 3.1. Experiment 1

#### Metabolizable Energy Prediction Equations

**Figure 1:**
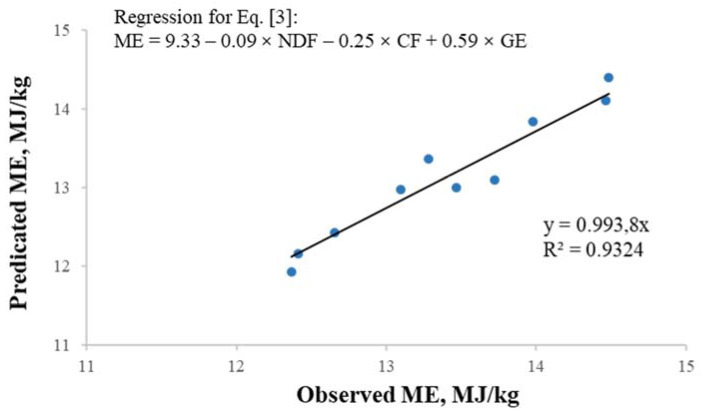
Relationships between predicted and observed values for metabolizable energy (ME) using Equation (3) (Experiment 1).

The stepwise regression equations and best prediction equations for ME in the DLRSC samples are summarized in Table 4. Neutral detergent fiber was the first predictor and CF was the second predictor. Considering the accuracy of measuring the ME, the equation that included NDF, CF, and GE was more practical with the greatest R^2^ (0.93) and the lowest AIC (−23.94) and RMSE (0.26). The best-fit equation to predict ME on a DM basis was ME (MJ/kg) = 9.33 − 0.09 × NDF − 0.25 × CF + 0.59 × GE. The comparison between the values predicted by the best-fit equation and the observed values are presented in Figure 1. The slope was almost 1 (R^2^ = 0.93; meaning the predicted values from the equation = observed values), which indicated great accuracy of the equation in predicting ME content. Therefore, chemical composition can be used to predict the ME in DLRSC when fed to growing pigs.

### 3.2. Experiment 2

#### 3.2.1. Growth Performance, Caloric Efficiency of ME, and Serum Parameters

The chemical compositions (%, DM basis) of DLRSC in Experiment 2 were in agreement with values for Experiment 1. According to the prediction equation obtained in Experiment 1, the calculated ME value of DLRSC in Experiment 2 was 13.58 MJ/kg (DM basis; Table 2). Increasing dietary DLRSC had no effect on initial BW, final BW, ADFI, ADG, and F/G. In the present experiment, the caloric efficiency method was used to verify the accuracy of the ME prediction equation. The caloric efficiency of ME (31.83, 32.44, 31.95, 32.69 MJ/kg, respectively) was not affected by increasing the concentration of DLRSC in diet (Table 5). Concentrations of T3 and T4 in serum decreased linearly (*p* < 0.05) as dietary levels of DLRSC increased.

#### 3.2.2. Nutrient Digestibility

Increasing dietary levels of DLRSC linearly reduced (*p* < 0.05) ATTD of DM, GE, CP, ADF, and OM of the diet (Table 6). There was a tendency (*p* = 0.056) for a linear decrease in ATTD of GE.

## 4. Discussion

Li et al. [6] determined the chemical compositions and ME content of ten DLRSC samples. Based on those data, ME prediction equations for DLRSC were developed in the present experiment. Previous studies successfully established prediction equations for energy content of flaxseed expellers [20], sorghum grains [21], barley [22], and corn [23]. The development of such prediction equations contributed to rapidly and accurately determining the energy content of feed ingredients. However, there is little information about prediction equations for DLRSC. To our knowledge, the results of the present study provide the first prediction equations for ME based on the measured chemical composition of DLRSC. More accurate prediction of energy values may improve diet formulation and reduce diet cost. In addition, due to the relatively complicated test process of the net energy evaluation system, the energy evaluation of feed ingredients is still based on digestive energy and ME.

It is not surprising that NDF and CF were the first and second predictors, because many reports have indicated that dietary fiber is a key factor affecting the energy content of a diet [24]. Higher fiber has been shown to reduce the energy content of DLRSC [6,25]. Gross energy was also one of the predictors of the best-fit prediction equation, which may be attributed to the fact that EE is a primary determinant of GE. Crude fat is not only a more digestible component in the intestine, but its presence can also improve the digestibility of other nutrients [26]. In this experiment, the optimal model to predict ME included three predictors: NDF, CF, and GE. However, a previous report indicated that the optimal model for predicting the ME value of canola meal, 00-rapeseed meal, and 00-rapeseed expellers fed to growing pigs was ME = –630.8 + 14.13 × ash + 5.02 × CF + 3.45 × ADF + 1.03 × digestible energy (R^2^ = 0.98) [27]. This difference may be due to the prediction equation established in the previous study being based on three rapeseed co-products, while the equation from the present study is based only DLRSC. The energy content of different rapeseed co-products varies greatly due to the variations in concentrations of nutrients in the seeds and differences in oil extraction procedures [27]. Therefore, it may be more accurate to base prediction equations of energy content on analyses of each rapeseed co-product.

Increasing the levels of dietary DLRSC had no effect on the growth performance of growing pigs, which agrees with results from studies with weaned pigs [4,5]. However, in contrast, there are reports that increasing dietary expeller-extracted canola meal content linearly decreased ADG and ADFI of growing pigs [28] and growing–finishing pigs [29]. These differences may be directly related to the concentrations of total glucosinolates in the diet. The DLRSC in the present Experiment 2 contained 9.38 μmol/g glucosinolates. Therefore, the calculated concentration of glucosinolates in the diet with 21% of DLRSC was 1.97 μmol/g diet, which was below the generally accepted glucosinolates tolerance level (2.0–2.5 μmol/g) for growing pigs [25,30]. However, the concentrations of total glucosinolates were 5.22 μmol/g in the diet containing 22.5% expeller-pressed canola meal [29] and 2.75 μmol/g in the diet containing 30% expeller extracted canola meal [28], which are greater than the highest glucosinolates tolerance level for growing pigs. In addition, increasing levels of extruded *B. juncea* expeller increased growth performance of weaned pigs due to a decrease in dietary net energy value and SID Lys/net energy ratio [31]. In general, glucosinolates in rapeseed co-products are considered as a limiting factor for their utilization in swine diets. However, when concentration of the dietary glucosinolates are below tolerance level, feeding DLRSC to replace soybean meal did not affect the growth performance of growing pigs fed diets balanced for SID Lys/ME ratio and SID AA systems.

Although the best ME prediction equation successfully fits the DLRSC samples used in the development of the model, there is no guarantee that ME values can be accurately predicted when this equation is applied to chemical composition data from other DLRSC samples. Therefore, validation of this equation is warranted. Some researchers have proposed the concept of caloric efficiency to verify the accuracy of the prediction equation. The assumption of this approach is that if the energy value assigned to a test ingredient is accurate, regardless of the ingredient inclusion level, a similar caloric efficiency will be calculated among diets [32,33]. Increasing the inclusion levels of dietary DLRSC in Experiment 2 had no effect on the caloric efficiency of ME, which indicated that the predicted ME value of DLRSC was accurate. In addition, concentrations of total glucosinolates in the diets were lower than the recognized maximum tolerance level, and dietary SID Lys/ME ratio and SID essential amino acids/SID Lys ratio were kept constant to meet requirements. Thus, energy value of diet may be the main factor affecting growth performance of pigs. Further, growth performance of pigs was not affected by increasing the levels of DLRSC, indicating that the actual ME values of four experimental diets were relatively consistent and close to the predicted ME values used in diet formulation. Therefore, the best-fit prediction equation obtained in Experiment 1 may be used to accurately calculate the ME value of DLRSC for growing pigs. In addition, prediction equations of energy content have been verified for dried distillers’ grains with solubles, using the cross-validation method [11], as well as for lipids and full-fat rice bran, using repeated experimentations [12,13]. Furthermore, some studies have determined the relative accuracy of ME prediction equations for de-oiled corn distillers’ dried grains with solubles [8] and corn [34] by comparing the effects of methods for calculations for metabolizable energy (prediction equation or nutrient composition table) of diet formulation on growth performance and carcass quality. However, each of these methods have advantages and shortcomings. There is no clear agreement on which method should be used to verify the accuracy of the prediction equation. Continued research is required to develop improved methods to verify the accuracy of the prediction equation.

Increasing dietary DLRSC reduced concentrations of T3 and T4 in serum in the present study, as also reported for pigs fed diets containing canola meal [35], expeller-extracted canola meal [28], and double-low rapeseed meal [36]. The break-down products of glucosinolates, such as oxazolidinethione and isothiocyanate, may impair function of the thyroid gland to decrease secretion of thyroid hormones [25,28]. Thyroid hormones are required for the normal growth and development of muscle, and their deficiency may inhibit growth of pigs [37]. However, in this experiment, the concentration of thyroid hormones in pigs fed DLRSC diets was not decreased sufficiently to reduce growth performance.

The ATTD values of nutrients in the diet decreased as DLRSC levels increased. Similarly, weaned pigs fed up to 200 g/kg expeller-pressed canola meal or canola press-cake or up to 240 g/kg extruded *Brassica juncea* expeller had lower nutrient digestibility values [4,5,31]. Furthermore, similar results were reported for pigs fed diets containing solvent-extracted meal [38]. The reduction in the ATTD of nutrients was likely due to the increase in fiber content as the dietary DLRSC levels increased. Greater dietary fiber intake can increase evacuation rate and decrease the transit time of nutrients in the intestine [39,40]. Increasing dietary fiber content may also increase endogenous excretion and decrease ATTD of nutrients [41]. In addition, decreases in nutrient digestibility may also be related to increases in concentrations of glucosinolates in the diet [38]. 

## 5. Conclusions

The best-fit prediction equation for accurately predicting ME (MJ/kg) for DLRSC to be fed to growing pigs is as follows: ME = 9.33 − 0.09 × NDF − 0.25 × CF + 0.59 × GE (R^2^ = 0.93). Increasing amounts of DLRSC in the diet linearly reduced ATTD of nutrients, but did not affect growth performance and caloric efficiency of ME of growing pigs fed diets balanced for SID Lys/ME ratio and SID AA systems.

## Figures and Tables

**Table 1:** Ingredient composition of experimental diets used in Experiment 1 (%, as-fed basis).

Items	Basal Diet	Test Diets ^4^
Corn	77.40	61.90
Soybean meal	18.60	14.90
Double-low rapeseed cake	-	19.22
Dicalcium phosphate	1.20	1.20
Limestone	1.10	1.10
Wheat rice stone ^1^	0.80	0.80
Salt	0.30	0.30
L-Lysine·HCl, 78% ^2^	0.10	0.08
Mineral and vitamin premix ^3^	0.50	0.50

^1^ Used as carrier for L-lysine·HCl, contained more than 70% silicon oxide and aluminum oxide, and produced by YiXian BeiQiao Tou Ore Company (YiXian, China).^2^ L-lysine·HCl was provided by Dacheng Group, Changchun, China.^3^ Vitamin/mineral premix provided the following per kg of complete diet for growing pigs: vitamin A, 5512 IU; vitamin D_3_, 2200 IU; vitamin E, 30 IU; vitamin K_3_, 2.2 mg; vitamin B_12_, 27.6 μg; riboflavin, 4.0 mg; pantothenic acid, 14.0 mg; niacin, 30.0 mg; choline chloride, 400.0 mg; folacin, 0.7 mg; thiamine 1.5 mg; pyridoxine 3.0 mg; biotin, 44.0 ug; Mn, 40.0 mg (MnO); Fe, 75.0 mg (FeSO_4_·H_2_O); Zn, 75.0 mg (ZnO); Cu, 100.0 mg (CuSO_4_·5H_2_O); I, 0.3 mg (KI); Se, 0.3 mg (Na_2_SeO_3_).^4^ Ten test diets contained 19.22% double-low rapeseed cake, which replaced 20% of the energy-supplying ingredients in the basal diet. Ten double-low rapeseed cake samples were provided by ten vegetable oil plants located in Hunan, Anhui, and Jiangxi Provinces of China [6].

**Table 2:** The analyzed chemical composition (%, DM basis) and predicted metabolizable energy (MJ/kg) of the double-low rapeseed cake used in Experiment 2 ^1^.

Items	Content
Chemical composition, %	
DM	96.34
GE, MJ/kg	21.03
CP	37.96
EE	10.25
CF	18.12
NDF	40.21
ADF	19.92
Ash	7.71
Ca	0.68
TP	0.99
TGS, μmol/g	9.38
Predicted ME, MJ/kg	
DM basis	13.58
As-fed basis	13.03

DM, dry matter; GE, gross energy; CP, crude protein; EE, ether extract; CF, crude fiber; NDF, neutral detergent fiber; ADF, acid detergent fiber; TP, total phosphorus; TGS, total glucosinolates; ME, metabolizable energy. ^1^ Calculated by the prediction equation ME = 9.33 − 0.09 × NDF −0.25 × CF + 0.59 × GE (R^2^ = 0.93, *p* < 0.001).

**Table 3:** The ingredients and nutrient levels of the experimental diets used in Experiment 2 (as-fed basis).

	Double-Low Rapeseed Cake, %			
Items	0	7	14	21
Ingredient composition, %				
Corn	75.44	75.38	75.31	75.25
Soybean meal	21.00	14.00	7.00	0.00
Double-low rapeseed cake, %	0.00	7.00	14.00	21.00
Dicalcium phosphate	1.20	1.14	1.13	1.14
Limestone	0.75	0.73	0.68	0.60
Salt	0.35	0.35	0.35	0.35
L-Lys·HCl, 78% ^1^	0.44	0.54	0.63	0.73
DL-Met	0.10	0.08	0.06	0.04
L-Trp	0.02	0.03	0.05	0.06
L-Thr	0.13	0.16	0.18	0.21
L-Val	0.07	0.09	0.11	0.12
Mineral and vitamin premix ^2^	0.50	0.50	0.50	0.50
Nutrient levels				
Analyzed composition, %				
DM	86.47	86.93	87.29	87.76
GE, MJ/kg	15.69	15.88	16.07	16.28
CP	15.40	15.27	14.91	14.17
NDF	15.99	16.39	18.18	18.64
ADF	6.72	6.98	7.63	8.10
Ash	4.33	4.35	4.48	4.32
Calculated composition				
ME, MJ/kg	13.83	13.78	13.72	13.66
SID Lys/ME	0.72	0.72	0.72	0.72
SID AA/SID Lys				
Met	0.33	0.32	0.31	0.30
Met + Cys	0.56	0.56	0.56	0.56
Trp	0.17	0.17	0.17	0.17
Thr	0.60	0.60	0.60	0.60
Val	0.65	0.65	0.65	0.65

DM, dry matter; GE, gross energy; CP, crude protein; NDF, neutral detergent fiber; ADF, acid detergent fiber; ME, metabolizable energy; SID, standardized ileal digestibility. ^1^ L-lysine hydrochloride was provided by the Dacheng Group, Changchun, China. ^2^ Vitamin/mineral premix provided the following per kg of complete diet for growing pigs: vitamin A, 5512 IU; vitamin D_3_, 2200 IU; vitamin E, 30 IU; vitamin K_3_, 2.2 mg; vitamin B_12_, 27.6 μg; riboflavin, 4.0 mg; pantothenic acid, 14.0 mg; niacin, 30.0 mg; choline chloride, 400.0 mg; folacin, 0.7 mg; thiamine 1.5 mg; pyridoxine 3.0 mg; biotin, 44.0 ug; Mn, 40.0 mg (MnO); Fe, 75.0 mg (FeSO_4_·H_2_O); Zn, 75.0 mg (ZnO); Cu, 100.0 mg (CuSO_4_·5H_2_O); I, 0.3 mg (KI); Se, 0.3 mg (Na_2_SeO_3_).

**Table 4:** Regression equations to estimate metabolizable energy (MJ/kg) in double-low rapeseed cake (Experiment 1) ^1^.

Number	Regression Equation	R^2^	AIC	RMSE	*p*-Value
1	ME = 18.32 − 0.12 × NDF	0.80	−17.15	0.39	<0.001
2	ME = 21.33 − 0.10 × NDF −0.21 × CF	0.88	−20.26	0.32	<0.001
3	ME = 9.33 − 0.09 × NDF − 0.25 × CF + 0.59 × GE	0.93	−23.94	0.26	<0.001

ME, metabolizable energy; NDF, neutral detergent fiber; CF, crude fiber; GE, gross energy; RSD, residual standard deviation; AIC, Akaike information criterion; RMSE, root mean square error. ^1^ Regression equations were developed by stepwise regression analyses.

**Table 5:** Effects of inclusion levels of double-low rapeseed cake on growth performance, serum parameters, and ME caloric efficiency in growing pigs (Experiment 2).

Items	Double Low Rapeseed Cake, %				SEM	*p*-Value	
	0	7	14	21		Linear	Quadratic
Initial BW, kg	29.74	29.62	29.72	29.73	1.20	0.992	0.958
Final BW, kg	51.15	50.65	50.56	49.82	1.73	0.601	0.942
ADFI, kg/d	1.76	1.76	1.73	1.72	0.07	0.608	0.972
ADG, kg/d	0.77	0.75	0.75	0.72	0.02	0.163	0.719
F/G	2.30	2.36	2.33	2.39	0.07	0.401	0.929
ME caloric efficiency, MJ/kg	31.83	32.44	31.95	32.69	1.00	0.607	0.941
T3, ng/mL	0.71	0.70	0.68	0.56	0.05	0.041	0.273
T4, ng/mL	47.73	42.67	39.19	29.72	2.30	<0.001	0.351

BW, body weight; ADFI, average daily feed intake; ADG, average daily gain; F/G, feed-to-gain ratio; ME, metabolizable energy; T3, triiodothyronine; T4, tetraiodothyronine.

**Table 6:** Effects of inclusion levels of double-low rapeseed cake on apparent total tract digestibility of nutrients in growing pigs (Experiment 2).

Items	Double-Low Rapeseed Cake, %				SEM	*p*-Value	
	0	7	14	21		Linear	Quadratic
DM	80.41	78.80	77.76	77.71	0.67	0.009	0.252
GE	78.82	77.26	76.44	76.40	0.80	0.041	0.356
CP	73.47	70.06	67.48	64.59	1.21	<0.001	0.831
NDF	55.23	52.24	52.03	52.17	1.98	0.302	0.437
ADF	54.30	51.62	50.10	46.94	1.63	<0.001	0.880
OM	84.04	82.74	81.90	81.68	0.59	0.009	0.372

DM, dry matter; GE, gross energy; CP, crude protein; NDF, neutral detergent fiber; ADF, acid detergent fiber; OM, organic matter.

## Data Availability

The data presented in this study are available from the corresponding author on request.

## Supplementary Materials

The following are available online at https://www.mdpi.com/article/10.3390/ani11041168/s1, Table S1: Chemical compositions and metabolizable energy values of double-low rapeseed cakes used in Experiment 1 (%, DM basis).
