# Supplementary material for: Development and Validation of Equations for Predicting the Metabolizable Energy Value of Double-Low Rapeseed Cake for Growing Pigs
Source: Animals (Basel). 2021 Apr 19;11(4):1168. doi: 10.3390/ani11041168 (PMC8073417; doi:10.3390/ani11041168)
Supplement: Supplementary file 1 [file animals-11-01168-s001.zip › animals-1176972-supplementary.pdf]

Supplementary

Table S1. Chemical compositions and metabolizable energy values of double-low rapeseed cakes used in Exp. 1 (%, DM basis) [1]

	Double-low rapeseed cakes									
Item	1	2	3	4	5	6	7	8	9	10
DM	95.82	92.25	92.41	90.28	92.65	92.33	90.79	92.17	93.45	91.84
GE, MJ/kg	21.20	20.94	21.41	21.66	21.08	20.88	21.45	20.90	20.66	20.70
CP	39.59	38.67	39.34	38.28	38.26	40.87	39.89	37.75	40.07	38.18
EE	8.38	10.44	10.23	11.14	10.72	9.27	9.84	10.76	8.75	9.83
NDF	53.78	49.00	37.91	34.95	37.28	39.89	45.02	48.15	41.76	39.22
ADF	30.15	29.46	22.84	23.38	23.54	25.22	27.72	26.97	24.53	22.99
CF	18.52	19.45	16.28	17.99	18.76	19.12	19.35	18.70	17.17	16.15
Ash	5.89	7.66	7.85	6.92	7.23	6.87	7.42	8.04	7.40	7.83
Ca	0.58	0.65	0.98	0.75	0.70	0.66	0.71	0.86	0.72	0.78
TP	0.83	1.02	1.20	1.02	1.22	1.04	1.06	1.18	1.05	1.17
TGS, μmol/g	7.40	10.00	5.40	28.90	14.30	23.70	9.90	11.60	17.80	21.00
ME, MJ/kg	11.93	12.16	14.41	14.11	13.10	13.37	12.98	12.43	13.00	13.84

DM, dry matter; GE, gross energy; CP, crude protein; EE, ether extract; NDF, neutral detergent fiber; ADF, acid detergent fiber; CF, crude fiber; TP, total phosphorus; TGS, total glucosinolates; ME, metabolizable energy

[1] Data of double-low rapeseed cakes were derived from published study in our laboratory [6].
